# The first documented interaction between a long‐tailed weasel (*Mustela frenata*) and a plains spotted skunk (*Spilogale interrupta*) carcass

**DOI:** 10.1002/ece3.9758

**Published:** 2023-01-29

**Authors:** Kara M. White, Joshua D. Stafford, Robert C. Lonsinger

**Affiliations:** ^1^ Department of Natural Resource Management South Dakota State University Brookings South Dakota USA; ^2^ U.S. Geological Survey, South Dakota Cooperative Fish & Wildlife Research Unit, Department of Natural Resource Management South Dakota State University Brookings South Dakota USA; ^3^ U.S. Geological Survey, Oklahoma Cooperative Fish & Wildlife Research Unit, Department of Natural Resource Ecology & Management Oklahoma State University Stillwater Oklahoma USA

**Keywords:** diet, Great Plains, interspecific interactions, long‐tailed weasel, plains spotted skunk

## Abstract

A novel interaction between a long‐tailed weasel (*Mustela frenata*) and a plains spotted skunk (*Spilogale interrupta*) carcass is detailed. In November 2020, a farmer in Edmunds County in north‐central South Dakota sent in a video recording of a long‐tailed weasel with a spotted skunk carcass. Location of the event, carcass condition, and recorded behavior of the long‐tailed weasel offer probable, but unconfirmed, evidence that the spotted skunk was killed by the long‐tailed weasel.

## Graphical abstract

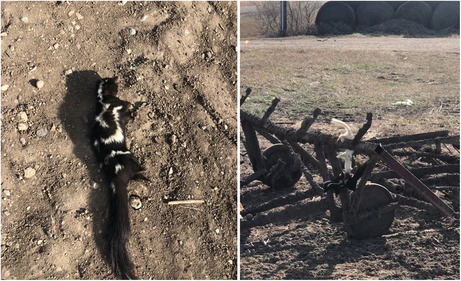

1

The first documented interaction between a long‐tailed weasel (*Mustela frenata*) and a plains spotted skunk carcass (*Spilogale interrupta*).

## 2 INTRODUCTION

Spotted skunks (*Spilogale* spp.) were historically distributed from southern Canada to Costa Rica and from the west coast of the contiguous United States to the Appalachian Mountains and Florida (Nilz & Finck, 2008). Spotted skunks have been associated with a variety of habitats that aggregate prey and provide thermal and escape cover including forests with brushy or rocky understories (Eng & Jachowski, 2019; Lesmeister et al., 2010; Reed & Kennedy, 2000; Sprayberry & Edelman, 2018), agricultural landscapes in proximity to farm buildings, grain elevators, corn cribs, and haybales (Crabb, 1948), and palmetto thickets (Kinlaw, 1995) in the subtropical dry prairie of Florida (Harris, Froehly, et al., 2021). An analysis of long‐term harvest trends indicated that spotted skunk populations east of the Rocky Mountains have declined >90% since the 1940s (Gompper & Hackett, 2005). Causes of this decline have not been identified, but plausible hypotheses include habitat change and loss via expansion of agriculture, widespread use of agricultural pesticides leading to decreased prey abundances, increased competition and changes in predator communities, historical overharvest, and disease (Gompper, 2017; Gompper & Hackett, 2005). Although spotted skunks east of the Rocky Mountains were historically considered a single species (the eastern spotted skunk; *S. putorius*), recent genetic analyses revealed significant divergence among previously recognized subspecies and indicated the plains (or prairie) spotted skunk should be classified as a separate species (*S. interrupta*; McDonough et al., 2022). The United States Fish and Wildlife Service is currently conducting a review of the conservation status of the plains spotted skunk in response to concerns over perceived population declines (Federal Register, 2012).

Research on spotted skunks has been limited, possibly because some consider them a nuisance species (Crabb, 1948; Kinlaw, 1995; Knight, 1994; Powell et al., 2017). Only 71 studies across *Spilogale* spp. were published from 1990 to 2019, of which 16 focused on plains spotted skunks. Information on spotted skunks in the Great Plains region is limited to a species description (Crabb, 1948), two species status evaluations (Choate et al., 1974; Tyler & Lodes, 1980), a report on incidental captures in South Dakota (Fino et al., 2019), and a description of weasel (*Mustela* spp.) and plains spotted skunk den use (Polder, 1968). Acknowledging the need for more information on the ecology of eastern and plains spotted skunks, researchers have begun addressing the knowledge gap by publishing >20 studies on eastern and plains spotted skunk in 2020 and 2021, but no new studies have occurred in the northern Great Plains.

Mephitids use various antipredator defense strategies when interacting with sympatric mammalian carnivores or birds of prey. Their aposematic pelage (Stankowich et al., 2011), coupled with feet stomping and tail lifting displays (Allen et al., 2013; Lartviere & Messier, 1996), serve as warnings of the noxious secretions they can express from their anal glands (Fisher & Stankowich, 2018). Potential competitors and predators may learn to avoid these visual and behavioral cues (Hunter, 2009); for example, Allen et al. (2013) described an encounter between a cougar (*Puma concolor*) and a western spotted skunk (*S. gracilis*), where the skunk successfully usurped and defended the cougar‐killed deer (*Odocoileus hemionus columbianus*) carcass. Although mephitids are not immune to interspecific killing, potential predators may avoid mephitids due to perceived risks or unpalatability (Allen, 2013; Wade‐Smith & Verts, 1982). Documented predators of mephitids include great horned owls (*Bubo virginianus*), bald (*Aquila chrysaetos*) and golden (*Haliaeetus leucocephalus*) eagles, cougars, coyotes (*Canis latrans*), badgers (*Taxidea taxus*), red (*Vulpes vulpes*) and gray (*Urocyon cinereoargenteus*) foxes, and bobcats (*Lynx rufus*) (Wade‐Smith & Verts, 1982). Despite the need for research on eastern and plains spotted skunks, little information exists about their interspecific interactions, and Lesmeister et al. (2010) identified predation as the primary cause of their mortality. Avian predators (e.g., barred owls (*Strix varia*; Hassler et al., 2021) and great horned owls (Lesmeister et al., 2010)) have been documented as spotted skunk predators. Native mammalian predators (e.g., wild canids and felids; Schwartz & Schwartz, 1981, Gompper, 2017) have not been formerly identified as predators of spotted skunks, but Crabb (1948) reported predation by domestic dogs (*Canis familiaris*) and cats (*Felis catus*).

The long‐tailed weasel (*M. frenata*) is one of the smallest members of the family Mustelidae (~170–280 g; Elsasser & Parker, 2008), whereas the plains spotted skunk is one of the smallest members of the family Mephitidae (~400–670 g; Fino et al., 2019, Higdon & Gompper, 2020). The long‐tailed weasel is a bold (Armitage, 1961) generalist predator that typically hunts small‐to‐medium‐sized prey (Sheffield & Thomas, 1997) but is capable of diet switching as relative prey abundances change (Proulx, 2019). Long‐tailed weasels employ many hunting strategies, actively hunting in search of prey in underground burrows, on the ground, and arboreally (Vaughan, 1961). Their most common diet items include small rodents, lagomorphs, and game birds (Polderboer et al., 1941), but long‐tailed weasels have been documented predating least (*M. nivalis*) and short‐tailed (*M. erminea*) weasels (Gamble, 1981), and big brown bats (*Eptesicus fuscus*; Mumford, 1969). Interspecific interactions have not been documented between plains spotted skunks and long‐tailed weasels, though in Iowa, both species commonly used Franklin's ground squirrel (*Poliocitellus franklinii*) and pocket gopher (*Geomys bursarius*) excavations as primary dens in areas with tall grass, which could instigate such interactions (Polder, 1968). Polder (1968) observed that spotted skunks frequently scavenged weasel caches, and Detweiler et al. (2021) documented a long‐tailed weasel investigating and briefly entering a spotted skunk den. Here, we present details of a potential predation of a plains spotted skunk by a long‐tailed weasel to further improve knowledge of potential interspecific interactions influencing plains spotted skunks.

## 3 RESULTS AND DISCUSSION

**FIGURE 1:**
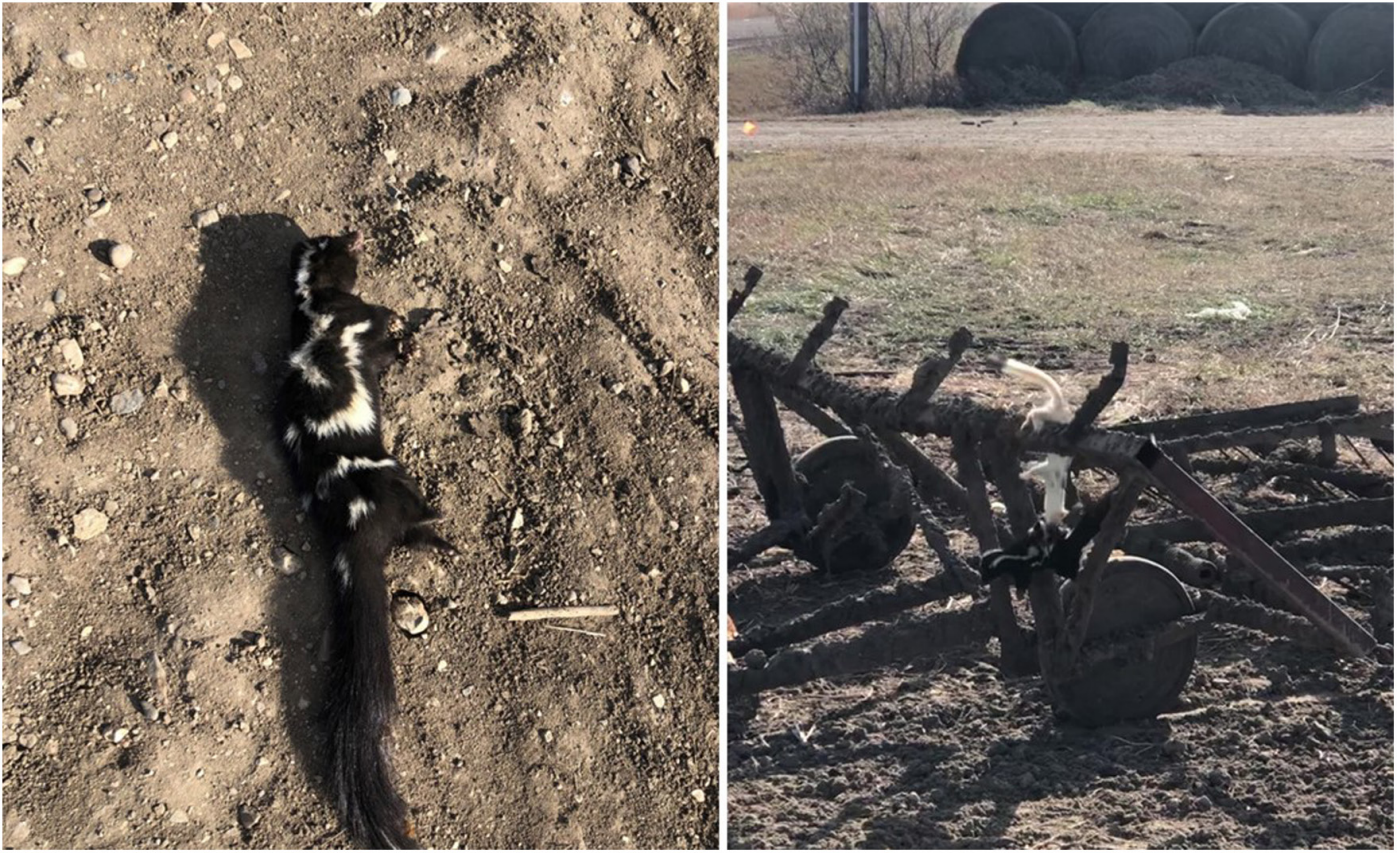
Plains spotted skunk carcass that was dropped by a long‐tailed weasel (left) after disturbance by humans and the long‐tailed weasel retrieving the carcass (right) after it was placed on farm equipment by observers on 12 November 2020 in South Dakota (images courtesy of Sean Christiansen of Edmunds County, South Dakota, used with permission).

On November 12, 2020, a farmer in north‐central South Dakota (Edmunds County) witnessed a weasel carrying the carcass of a spotted skunk in an area where the landowner stored hay bales and farm equipment. The weasel dropped the carcass and retreated upon noticing the observer. The farmer then took photographs of the spotted skunk carcass (Figure 1), placed it on top of a small piece of farming machinery, backed away to allow the weasel to return and retrieve the carcass, and recorded the interaction using his cellular phone camera. In December 2020, the farmer provided images and a 64‐s video of the interaction, which is described herein. Initially, the weasel approached and repeatedly circled the farm machinery where the spotted skunk was placed. The weasel eventually climbed to the carcass, bit it, dropped to the ground with the carcass in its mouth, and retreated with the carcass to nearby hay bales. The images and video were used to confirm the carcass as a spotted skunk and the predator as a long‐tailed weasel. The video is embedded (Video 1) and archived (https://openprairie.sdstate.edu/nrm_studentwork/1/).

The interaction may indicate the weasel killed the skunk or was scavenging skunk carrion. Long‐tailed weasels may scavenge (DeVault et al., 2011; Elbroch et al., 2017), but there is no evidence of weasels scavenging a mephitid carcass. However, the location of the encounter and condition of the carcass suggests that the spotted skunk was likely killed by the weasel. The interaction occurred in a rural county (i.e., 1.4 people and 0.66 housing units/km^2^; United States Census Bureau, 2020) that was almost entirely farmland (~99.9%; USDA, 2017). The site where the interaction occurred received little human traffic and no paved roads were within ~18 km, reducing the potential that the weasel scavenged a roadkill. Furthermore, no physical signs of injury associated with a vehicle collision were observed, and the carcass did not have signs of rigor mortis, indicating the spotted skunk was recently deceased (Harris, Olfenbuttel, & Jachowski, 2021). It is possible, though not confirmed, that spotted skunk mortality could result from ingesting rodenticides near home and farm buildings, but the nearest farm building was substantially further from the interaction site (~1.3 km) than the reported maximum winter travel distance (465 m; DeVan, 1982) or expected movement rates for long‐tailed weasels based on home range sizes in agricultural landscapes (Gehring & Swihart, 2004). It is also unlikely that the long‐tailed weasel usurped prey from another predator given that they are among the smallest predators in the carnivore guild, and predators of spotted skunks are capable of killing weasels; therefore, any attempt to scavenge from a shared predator would risk injury or death of the weasel (Palomares & Caro, 1999). Finally, the carcass was not partially consumed, as would be expected if it had been scavenged following predation by another species. Consequently, the evidence suggests that the recorded interaction was the result of a predatory killing of a spotted skunk by a long‐tailed weasel.

## AUTHOR CONTRIBUTIONS

**Kara M. White:** Conceptualization (equal); writing – original draft (lead); writing – review and editing (equal). **Joshua Stafford:** Writing – review and editing (equal). **Robert Lonsinger:** Conceptualization (equal); writing – review and editing (equal).

## CONFLICT OF INTEREST

The authors declare that there is no conflict of interest.

## Data Availability

Data sharing not applicable to this article as no datasets were generated or analysed during the current study.
